# A Genome-Wide Scan of Ashkenazi Jewish Crohn's Disease Suggests Novel Susceptibility Loci

**DOI:** 10.1371/journal.pgen.1002559

**Published:** 2012-03-08

**Authors:** Eimear E. Kenny, Itsik Pe'er, Amir Karban, Laurie Ozelius, Adele A. Mitchell, Sok Meng Ng, Monica Erazo, Harry Ostrer, Clara Abraham, Maria T. Abreu, Gil Atzmon, Nir Barzilai, Steven R. Brant, Susan Bressman, Edward R. Burns, Yehuda Chowers, Lorraine N. Clark, Ariel Darvasi, Dana Doheny, Richard H. Duerr, Rami Eliakim, Nir Giladi, Peter K. Gregersen, Hakon Hakonarson, Michelle R. Jones, Karen Marder, Dermot P. B. McGovern, Jennifer Mulle, Avi Orr-Urtreger, Deborah D. Proctor, Ann Pulver, Jerome I. Rotter, Mark S. Silverberg, Thomas Ullman, Stephen T. Warren, Matti Waterman, Wei Zhang, Aviv Bergman, Lloyd Mayer, Seymour Katz, Robert J. Desnick, Judy H. Cho, Inga Peter

**Affiliations:** 1 Department of Computer Sciences, Columbia University, New York, New York, United States of America; 2 Department of Gastroenterology, Rambam Health Care Campus, B. Rappaport Institute for Research in the Medical Sciences, Faculty of Medicine, Technion-Israel Institute of Technology, Haifa, Israel; 3 Department of Genetics and Genomic Sciences, Mount Sinai School of Medicine, New York, New York, United States of America; 4 Department of Medicine, Section of Digestive Diseases, Yale University, New Haven, Connecticut, United States of America; 5 Department of Pathology, Albert Einstein College of Medicine, New York, New York, United States of America; 6 Division of Gastroenterology, University of Miami Miller School of Medicine, Miami, Florida, United States of America; 7 Genetic Core for Longevity, Institute for Aging Research and the Diabetes Research Center, Albert Einstein College of Medicine, Bronx, New York, United States of America; 8 Meyerhoff Inflammatory Bowel Disease Center, Department of Medicine, Johns Hopkins University School of Medicine, Baltimore, Maryland, United States of America; 9 Department of Epidemiology, Bloomberg School of Public Health, Johns Hopkins University, Baltimore, Maryland, United States of America; 10 Mirken Department of Neurology, Beth Israel Medical Center, New York, New York, United States of America; 11 The Saul R. Korey Department of Neurology, Albert Einstein College of Medicine, Bronx, New York, United States of America; 12 Department of Medicine, Albert Einstein College of Medicine, Bronx, New York, United States of America; 13 Department of Pathology and Cell Biology, Columbia University, New York, New York, United States of America; 14 The Institute of Life Sciences, Hebrew University of Jerusalem, Jerusalem, Israel; 15 Division of Gastroenterology, Hepatology, and Nutrition, Department of Medicine, University of Pittsburgh School of Medicine, Pittsburgh, Pennsylvania, United States of America; 16 Department of Human Genetics, Graduate School of Public Health, University of Pittsburgh, Pittsburgh, Pennsylvania, United States of America; 17 Department of Gastroenterology and Hepatology, Sheba Medical Center, Raman Gan, Israel; 18 Department of Neurology, Tel Aviv Sourasky Medical Center, Sackler School of Medicine, Tel Aviv University, Tel-Aviv, Israel; 19 Robert S. Boas Center for Genomics and Human Genetics, Feinstein Institute for Medical Research, North Shore LIJ Health System, Manhasset, New York, United States of America; 20 Center for Applied Genomics, The Children's Hospital of Philadelphia, University of Pennsylvania School of Medicine, Philadelphia, Pennsylvania, United States of America; 21 Division of Endocrinology, Diabetes, and Metabolism, Graduate Program in Biomedical Sciences and Translational Medicine, Cedars-Sinai Medical Center, Los Angeles, California, United States of America; 22 Department of Neurology, College of Physicians and Surgeons, Columbia University, New York, New York, United States of America; 23 Taub Institute for Research on Alzheimer's Disease and the Aging Brain, College of Physicians and Surgeons, Columbia University, New York, New York, United States of America; 24 Department of Translational Medicine, Inflammatory Bowel and Immunobiology Research Institute, Cedars-Sinai Medical Center, Los Angeles, California, United States of America; 25 Medical Genetics Institute, Cedars-Sinai Medical Center, Los Angeles, California, United States of America; 26 Department of Human Genetics, Emory University School of Medicine, Atlanta, Georgia, United States of America; 27 Genetic Institute, Tel Aviv Sourasky Medical Center, Sackler School of Medicine, Tel Aviv University, Tel-Aviv, Israel; 28 Epidemiology-Genetics Program in Schizophrenia, Bipolar Disorders, and Related Disorders, Department of Psychiatry and Behavioral Sciences, School of Medicine, Johns Hopkins University, Baltimore, Maryland, United States of America; 29 Department of Epidemiology, Bloomberg School of Public Health, Johns Hopkins University, Baltimore, Maryland, United States of America; 30 IBD Group, Mount Sinai Hospital, University of Toronto, Toronto, Canada; 31 Division of Gastroenterology, Department of Medicine, Mount Sinai School of Medicine, New York, New York, United States of America; 32 Departments of Biochemistry and Pediatrics, Emory University School of Medicine, Atlanta, Georgia, United States of America; 33 Department of Systems and Computational Biology, Albert Einstein College of Medicine, New York, New York, United States of America; 34 Albert Einstein College of Medicine, North Shore University Hospital-Long Island Jewish Hospital Systems, St. Francis Hospital, Great Neck, New York, United States of America; University of Michigan, United States of America

## Abstract

Crohn's disease (CD) is a complex disorder resulting from the interaction of intestinal microbiota with the host immune system in genetically susceptible individuals. The largest meta-analysis of genome-wide association to date identified 71 CD–susceptibility loci in individuals of European ancestry. An important epidemiological feature of CD is that it is 2–4 times more prevalent among individuals of Ashkenazi Jewish (AJ) descent compared to non-Jewish Europeans (NJ). To explore genetic variation associated with CD in AJs, we conducted a genome-wide association study (GWAS) by combining raw genotype data across 10 AJ cohorts consisting of 907 cases and 2,345 controls in the discovery stage, followed up by a replication study in 971 cases and 2,124 controls. We confirmed genome-wide significant associations of 9 known CD loci in AJs and replicated 3 additional loci with strong signal (p<5×10^−6^). Novel signals detected among AJs were mapped to chromosomes 5q21.1 (rs7705924, combined p = 2×10^−8^; combined odds ratio OR = 1.48), 2p15 (rs6545946, p = 7×10^−9^; OR = 1.16), 8q21.11 (rs12677663, p = 2×10^−8^; OR = 1.15), 10q26.3 (rs10734105, p = 3×10^−8^; OR = 1.27), and 11q12.1 (rs11229030, p = 8×10^−9^; OR = 1.15), implicating biologically plausible candidate genes, including *RPL7*, *CPAMD8*, *PRG2*, and *PRG3*. In all, the 16 replicated and newly discovered loci, in addition to the three coding *NOD2* variants, accounted for 11.2% of the total genetic variance for CD risk in the AJ population. This study demonstrates the complementary value of genetic studies in the Ashkenazim.

## Author Summary

Crohn's disease causes inflammation of the digestive tract resulting from the interaction of normal gut bacteria with the host immune system in genetically predisposed individuals. People of Jewish heritage have an increased risk of developing Crohn's disease compared to non-Jewish Europeans. So far, 71 genetic variants that increase the risk of Crohn's disease have been identified in individuals of European ancestry. Here, we take advantage of recent technical and methodological advances to explore Crohn's diseases-related genetic variants specific to the Ashkenazi Jewish population. We examined 6,347 individuals whose Jewish ancestry was confirmed by a large number of genetic markers and detected several variants associated with the increased risk of Crohn' disease. We confirmed the involvement of 12 known Crohn's disease risk variants in Ashkenazi Jews and identified novel genetic regions not previously found in non-Jewish European populations. Further studies of these regions may help discover biological pathways affecting susceptibility to Crohn's disease and lead to the development of novel treatments. This study also demonstrates the complementary value of genetic studies in isolated populations, like the Ashkenazim.

## Introduction

Ashkenazi Jews (AJs) comprise a single genetic community of individuals of Eastern and Central European descent. Several lines of evidence suggest genetic differences between the Jewish and non-Jewish peoples of Europe (NJ). It has been demonstrated that the genomes of individuals with one to four grandparents of Jewish descent carry an unambiguous signature of their heritage allowing a perfect inference of their Jewish ancestry [1]. When studied separately, Jewish populations represent a series of geographical clusters with each group demonstrating Middle Eastern ancestry and variable admixture with European populations [2], [3]. Moreover, Price et al. [4] have shown that AJ ancestry is one of the major determinants of population structure amongst disease groups of European Americans and can be easily discerned by a small panel of genetic markers.

Genetic differences between Jewish and non-Jewish populations have been detected in the context of multiple monogenic conditions that are more prevalent in AJ populations. More than 25 recessive disease founder alleles have been found to afflict Ashkenazi populations at much elevated frequencies [5], [6] compared to NJ populations, resulting in a higher incidence of rare disorders including Tay Sachs disease, Canavan, Niemann-Pick, Gaucher, and others. Considerably higher frequencies of particular mutations strongly associated with common diseases, such as breast cancer (*BRCA1* 185delAG) [7] and Parkinson's disease (*LRRK2* G2019S) [8] have also been detected in AJ compared to NJ. Moreover, a three-phase genome-wide association study (GWAS) conducted in an AJ population has identified a novel region on 6q22.33 associated with familial breast cancer risk [9].

Crohn's disease (CD) is an inflammatory bowel disease resulting from dysregulated mucosal immune responses to enteric microbiota which arise in genetically susceptible individuals (reviewed in [10]). CD is 2–4 times more prevalent among AJs compared to NJ populations [11], [12]. Association scans in predominantly NJ CD studies have identified 71 susceptibility loci associated with the disease risk including coding polymorphisms at *NOD2*, *IL23R*, *ATG16L1* and an intergenic region on chromosome 5p13 [13], [14], [15], [16], [17], [18]. In our recent work, we showed that genetic risks associated with CD in the AJ population for the 22 most frequently replicated variants were similar to those reported in NJ populations [19] and, therefore, are unlikely to explain the excess disease prevalence in individuals of AJ descent. Although underlying mechanisms responsible for ethnicity-specific differences may include epigenetic and environmental factors, it has been hypothesized that substantially increased risk of CD in AJ versus NJ can be explained through the involvement of yet unknown genetic variants predominantly in this population. Therefore, the goal of the present study was to conduct a comprehensive GWAS to identify AJ-specific loci that predispose to CD, by testing for association in participants of self-identified and genetically verified AJ ancestry across multiple collections of cases and controls.

## Results

### Confirming Ashkenazi ancestry of study participants

**Figure 1:**
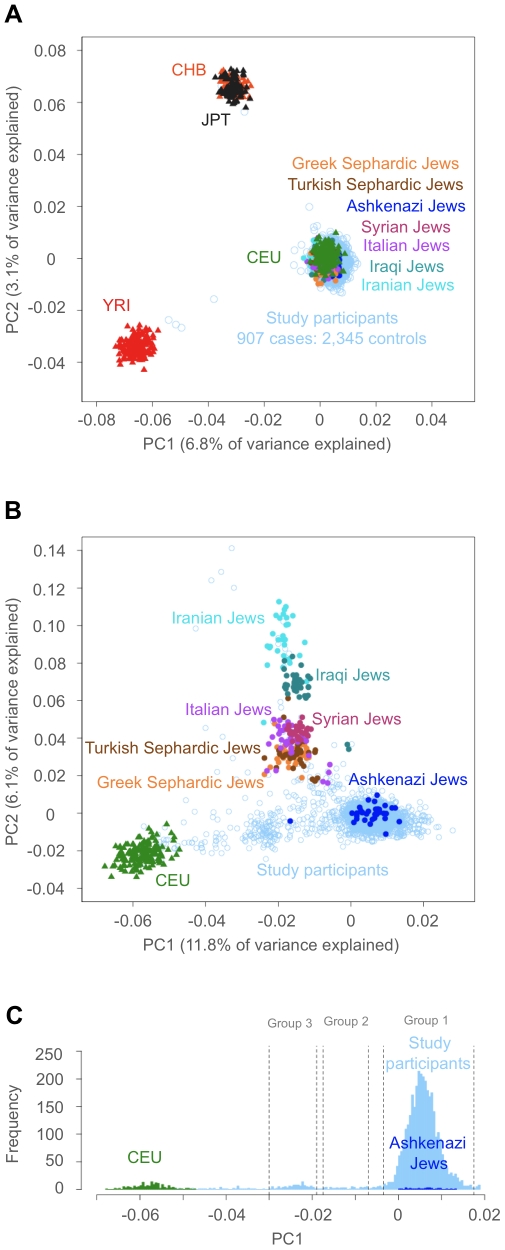
PCA analysis of the study participants. (A) PCA showing the first (X-axis) and second (Y-axis) eigenvectors plotting all 3,252 study participants (907 CD cases and 2,345 controls) across ∼22 K unlinked SNPs indicated by light blue open circles. Also included and color-coded in the graph are the four HapMap (www.hapmap.org) reference samples as solid triangles; CEPH-Utah (CEU; green), Yoruban-Nigeria (YRI; red), Han Chinese (CHB; orange) and Japanese (JPT; dark grey); and seven Jewish samples from the Jewish Hapmap project [2] as solid circles, consisting of Ashkenazi Jews (AJ; blue), one European (Italian; purple), three Middle Eastern (Syrian; fuchsia, Iraqi; teal and Iranian; turquoise) and two Sephardic Jewish cohorts (Turkey; brown and Greek; orange). (B) The same analysis excluding the YRI and CHB+JPT reference panels. (C) A histogram of PC1 values for study participants (light blue) near the AJ cluster and intermediate between the AJ and CEU clusters. The histogram of PC1 values for the included samples show three distinct modes (Groups 1–3), with AJ reference (blue) and CEU (green) indicated.

The population under examination in this study is a genetically distinct group in terms of ancestry, thus it was especially important to verify the genetic AJ ancestry of the study participants in the discovery stage. We performed PCA to determine the main axis of variation explaining the study cohort data. Results of the principal component analysis (PCA), plotting the samples with the three continental HapMap reference panels (European; CEU, African; YRI and Asian; CHB and JPT) and seven panels from the Jewish HapMap consortium consisting of one Ashkenazi Jewish, one European Jewish, three Middle Eastern and two Sephardic Jewish panels, are shown on Figure 1A. As expected, the first principal component (PC 1) distinguishes Africans from non-Africans and PC 2 distinguishes East Asians from Africans and individuals of European and Jewish ancestry (Figure 1A). Close examination of within-continent variation was performed by repeating this analysis excluding the CHB, JPT and YRI samples. Here we show that PC 1 distinguished European from Jewish ancestry (Figure 1B) and PC 2 shows a Middle Eastern to European cline of Jewish populations, with the majority of AJ individuals (∼80%) clustering distinctly from other European Jewish populations. Most of the remaining AJ samples (n = ∼500) are intermediate on a PC 1 cline between the AJ cluster and the European (CEU) cluster (Figure 1B). Upon examining the distribution of PC1 values in these samples, three distinct modes were defined; Group 1 (PC1<−0.005), Group 2 (PC1 −0.039- −0.046) and Group 3 (PC1 −0.036- −0.019) (Figure 1C). We postulated, based on previous PCA analysis of AJ individuals that groups 2 and 3 might represent individuals with 75% (one non-AJ grandparent) and 50% (one non-AJ parent or two non-AJ grandparents) AJ ancestry, respectively (Table S1). To avoid exclusion of individuals with partial AJ ancestry, we performed association mapping within each group independently to control for admixture effects, and combined the p-values from each group under a meta-analysis design to construct a single test statistic.

### Genome-wide association mapping of CD in AJ population

**Figure 2:**
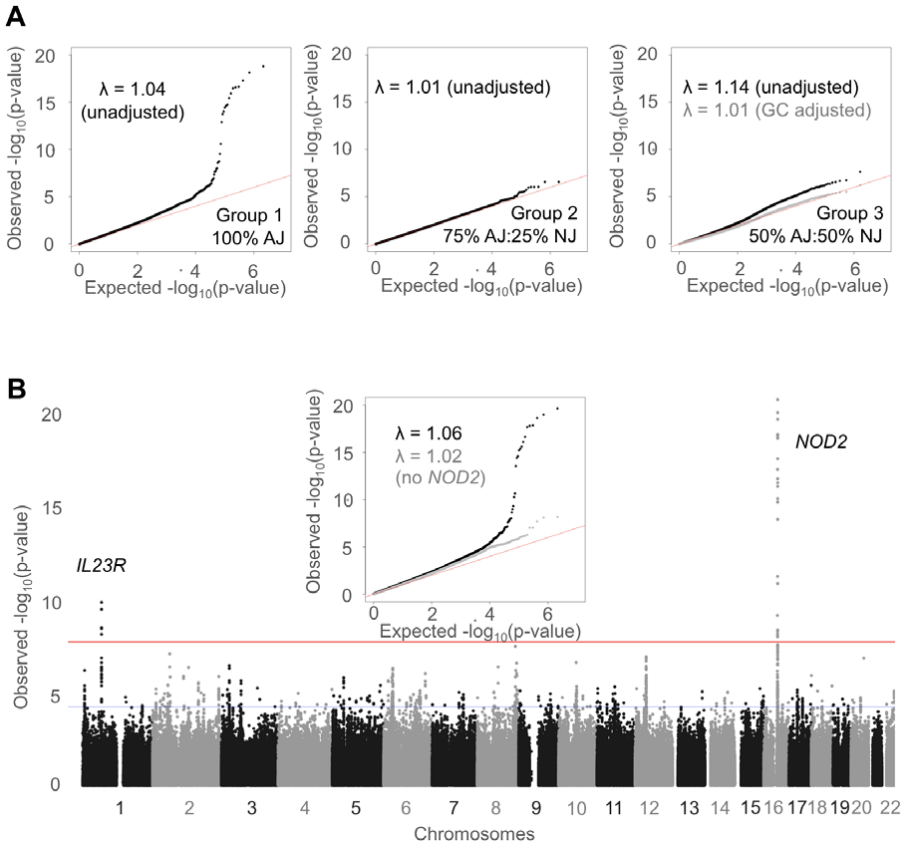
Association mapping of Crohn's disease in Ashkenazi Jews. (A) QQ-plots of the 100%, 75% and 50% AJ ancestry groups (Groups 1, 2 and 3, respectively). The inflation factors for the p-value distributions are given. For group 3, the p-values were genomic control-adjusted for over-inflation. (B) A Manhattan plot and QQ-plot (inset – in black) of the combined association scores from all three groups. The genome-wide threshold is shown in red and the replication threshold is shown in blue. The QQ-plot also shows association scores from all three groups but with 298 markers around the region of the *NOD2* signal on chromosome 16 removed before association mapping (in grey).

Details of the initial discovery GWAS panels and an independent AJ replication panel as well as the genotyping platforms used are given in Table 1. The final filtered dataset used for association mapping comprised 1,060,934 genotyped and imputed markers across 3,016 individuals (Figure S1). The dataset was divided into three groups according to AJ ancestry (Figure 1C). Figure 2A shows the QQ-plots for 100%, 75% and 50% AJ ancestry groups (groups 1, 2 and 3, respectively). In the case of group 3, the p-values were overinflated (λ = 1.14) and were corrected by genomic control to approximate normality uniform distribution [20]. Figure 2B shows the combined score from all three groups. Two known CD loci exceed the genome-wide significance threshold: *NOD2* (16q12; rs2076756; p<2.32×10^−20^) and *IL23R* (1p31; rs11209026; p<9.42×10^−9^) [14], [15], [16], [21]. In addition, 11 other previously reported CD signals at p<10^−4^ were *PUS10/REL* (2p16.1; rs13003464; p<1.98×10^−7^), *ATG16L1* (2q37; rs2241880; p<2.88×10^−6^), intergenic region >300 kb upstream of *PTGER4* (5p13.1; rs9292777; 6.92×10^−5^), *IL3* (5q31; rs3091338; p<4.86×10^−5^), *HLA* region (6p22.1; rs9258260), an intergenic region on 8q24.13 (p<9.25×10^−5^), *JAK2* (9p24.1;rs2230724; p<8.11×10^−5^), *ZNF365* (10q21; rs1076165; p<1.86×10^−5^), *NKX2-3* (10q24.2; rs11190141; 9.8×10^−5^), *PSMB10* (16q22.1; rs11574514; p<8.05×10^−5^) and *CCL7/CCL2* (17q12; rs3091316; 1.93×10^−5^) [13], [17]. The full set of SNPs showing association signal at a level of p<10^−4^ includes 616 SNPs across 137 distinct regions. Finally, since strong signals are prone to skew p-value distribution and can cause over-dispersion, especially at the tail, we assessed the p-value distribution with and without *NOD2* SNPs. The signal at these loci persists even after controlling for the strong signal at *NOD2* (Figure 2B inset).

**Table 1:** Study cohort description.

Data Source	N_total_	N_CD cases_	N_non-CD cases_	N_controls_	Platform
**Discovery GWAS Panel**					
NIDDK IBD genetics consortium^a^	828	397	-	431	Illumina 300 k
Pediatrics IBD Consortium^a^	136	136	-	-	Illumina 550 k
Mount Sinai School of Medicine I	173	113	-	60	Affymetrix 500 k
Mount Sinai School of Medicine II	532	261	271	-	Affymetrix 6.0
John Hopkins University	535	-	535	-	Affymetrix 6.0
Albert Einstein College of Medicine	651	-	-	651	Affymetrix 6.0
Hebrew University of Jerusalem	397	-	200	197	Illumina 300 k
**Total**	**3,252**	**907**	**1,006**	**1,339**	
**Replication Panel**					
Cedars-Sinai Medical Center^b^	348	348	**-**	**-**	Illumina 610 k
NIDDK IBD genetics consortium	59	59	-	-	Illumina 1 M
Columbia University	444	-	267	177	Illumina 660 k
Tel Aviv Sourasky Medical Center	426	-	298	128	Affymetrix 6.0
Mount Sinai School of Medicine	1,254	203	-	1051	Sequenom/iPLEX
Connecticut-Long Island	142	142	-	-	Sequenom/iPLEX
Rambam Medical Center	445	239	-	203	Sequenome/iPLEX
**Total**	**3,118**	**971**	**565**	**1,559**	

For each screen, the total number of individuals examined is shown (N_total_), in addition to any Crohn's disease cases (NC_D_cases_), non-Crohn's disease cases, which are a mix of individuals with Parkinson's disease, Schizophrenia, Type-2 Diabetes and Dystonia, (N_non-CD_cases_), and non-diseased controls (N_controls_).

b Genotypes available for only a subset of 31 replication markers.

### Replication studies in independent AJ samples identify five novel regions associated to CD

We followed a region-centric strategy for replication. If a single marker exceeded p<1×10^−4^ in a “signal region” (defined by the furthest up- and down-stream SNP in linkage disequilibrium (LD) with the marker, r^2^>0.2), it was included in the replication dataset. In the cases where a region contained multiple markers with p<1×10^−4^, 1–7 tag SNPs were selected from the region. The final set of replication markers comprised 175 SNPs across 137 regions, 139 of which were successfully genotyped in the replication dataset (see Table S2). Applying a standard genome-wide significance threshold of 5×10^−8^ for the combined discovery and replication signals, we observed 21 SNPs that surpassed this threshold in 14 distinct genetic regions (Table 2).

**Table 2:** Regions identified in the Ashkenazi Jewish CD GWAS, replication, and combined association analyses.

SNP	Cytogenetic location	Position (MB)^a^	Candidate Genes^b^(by genetic location ±250 kb)	Risk Allele (Freq)^c^	OR(95% CI)^d^	Discoveryp-value^e^	Replicationp-value^f^	Combinedp-value^g^
Association of novel gene regions:								
rs6545946	2p15	62.57	*TMEM17, EHBP1, ♦CPAMD8(5.7), ♦AK3(5.5)*	C (0.770)	1.16(1.06–1.27)	3.03×10^−6^	2.31×10^−3^	7.01×10^−9^
rs7705924	5q21.1	101.98	*SLCO6A1*	G (0.066)	1.48(1.17–1.87)	3.77×10^−5^	4.72×10^−4^	1.78×10^−8^
rs12677663	8q21.11	74.17	*C8orf84, TERF1, RPL7, RDH10, KCNB2*	T (0.659)	1.15(1.04–1.28)	9.46×10^−7^	2.08×10^−2^	1.96×10^−8^
rs10734105	10q26.3	133.06	*TCERG1L*	G (0.375)	1.27(1.10–1.43)	9.80×10^−5^	3.41×10^−4^	3.34×10^−8^
rs11229030	11q12.1	56.96	Multiple, including *SLC43A3, PRG2, PRG3*	C (0.305)	1.15(1.10–1.39)	4.01×10^−5^	2.11×10^−4^	8.45×10^−9^
Association of previously known regions at genome-wide significance:								
rs11209026	1p31.3	67.48	***IL23R*** *, IL12RB2, C1orf141, SERBP1, SLC35D1*	G (0.921)	2.20(2.10–2.35)	9.42×10^−9^	1.59×10^−10^	1.49×10^−18^
rs13003464	2p16.1	61.04	***PUS10*** *, PEX13, REL, KIAA1841, C2orf74, PAPOLG, USP34*	G (0.487)	1.05(1.00–1.40)	1.98×10^−7^	2.4×10^−2^	4.73×10^−9^
rs2241880	2q37.1	233.84	***ATG16L1*** *, SAG, DGKD, INPP5D, USP40*	G (0.601)	1.32(1.24–1.41)	2.88×10^−6^	5.02×10^−7^	1.44×10^−12^
rs9292777	5p13.1	40.31	*PTGER4*	T (0.597)	1.37(1.28–1.48)	6.92×10^−5^	9.12×10^−7^	2.13×10^−11^
rs3091338	5q31.1	131.43	*IL3, ACSL6, P4HA2, PDLIM4, SLC22A4*	T (0.328)	1.23(1.08–1.42)	4.86×10^−5^	9.20×10^−4^	4.47×10^−8^
rs9258260	6p22.1	29.83	*HLA-F, MOG, HLA-G, GABBR1, HLA-H, UBD, HLA-A*	T (0.104)	1.45(1.21–1.68)	2.19×10^−5^	7.92×10^−6^	1.74×10^−10^
rs7076156	10q21.2	64.09	***ZNF365*** *, ERG2, ADO*	G (0.751)	1.19(1.10–1.30)	1.86×10^−5^	3.91×10^−4^	7.29×10^−9^
rs2076756	16q12.1	49.3	***NOD2*** *, CYLD, SNX20, NKD1*	G (0.246)	1.66(1.48–1.88)	2.32×10^−20^	5.87×10^−18^	1.36×10^−37^
rs3091316	17q12	29.62	*CCL7, CCL2, CCL11, CCL8, CCL13, CCL1*	G (0.732)	1.14(1.03–1.27)	1.93×10^−5^	2.02×10^−3^	3.89×10^−8^
Replication of previously known regions:								
rs1906493	8q24.13	127.16	Intergenic	A (0.432)	1.19(1.09–1.28)	9.25×10^−5^	3.18×10^−2^	2.94×10^−6^
rs11190141	10q24.2	101.28	*NKX2-3, SLC25A28, GOT1, ENTPD7, CNNM1, COX15, CUTC*	C (0.739)	1.34(1.25–1.43)	9.80×10^−5^	5.19×10^−3^	5.08×10^−7^
rs11574514	16q22.1	66.53	Multiple, including *PSMB10*	A (0.045)	1.44(1.35–1.52)	8.05×10^−5^	2.56×10^−3^	2.06×10^−7^

a Physical position in megabases; Genome build NCBI36/hg18.

b Genes highlighted by genetic location of the top SNP ±250 kb, ordered by proximity to the top SNP. If the top SNP is intragenic, the gene is indicated in bold font. Additionally, if there is evidence of eQTL effect of LOD≥5 this is indicated with a *♦* symbol and the LOD is given in brackets.

c The risk allele in the AJ cohort with its frequency in healthy controls given in parenthesis.

d The odds ratio for the risk allele in the replication cohort, with ±95% confidence intervals given in parenthesis.

e,f,g p-values for the initial discovery GWAS for Crohn's disease in Ashkenazi Jews (Discovery p-value), replication cohort (Replication p-value) and a combined score of both p-values (Combined p-value) are given. Association significance thresholds are 5×10^−8^, 0.05, and 5×10^−8^ for discovery, replication and combined p-values, respectively. The significance thresholds of gene regions previously associated in other cohorts are 5×10^−6^, 0.05 and 5×10^−6^ for discovery, replication and combined p-values, respectively.

**Figure 3:**
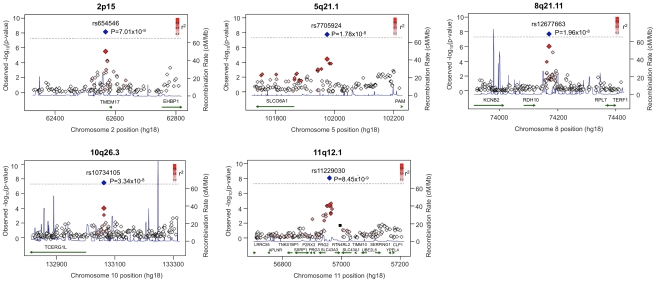
Regional plots of five novel associations to Crohn's disease in Ashkenazi Jews. Regional plots of the SNP p-values obtained in the discovery GWAS for a ±250 kb window around each of the 5 novel SNPs. The X-axis shows the chromosome and physical distance (kb), the left Y-axis shows the negative base ten logarithm of the p-value and the right y-axis shows recombination activity (cM/Mb) as a blue line. The chromosomal band is given above each plot. The replication SNP is indicated as a large red diamond, and linkage disequilibrium of surrounding SNPs with the replication SNP is indicated by a scale of intensity of red color filling as shown in the legend at the upper right hand corner of each plot. The combined discovery and replication p-value for the replication SNP is shown in blue, and is annotated with the SNP identifier and combined p-values. The position and location of any copy number variation in the mapping intervals are shown as a black rectangle. Positions, recombination rates and gene annotations are according the NCBI's build 36 (hg 18).

As positive controls, we report 9 of the 13 known loci listed in the previous section exceeding our threshold of association in the AJ population, with a further three surpassing our replication threshold for known regions of association to CD, 5×10^−6^ (Table 2 and Figure S2). Furthermore, novel signals of association in the AJ sample were observed for five regions not previously reported. Regional association plots of all five novel regions are shown in Figure 3 and their risk allele frequencies and odd ratios (ORs) are shown in Table 2. Two of these regions (5q21.1; rs7705924; 1.78×10^−8^ and 10q26.3; rs10734105; 3.34×10^−8^) contained just a single gene, *SLCO6A1* and *TCERG1L*, respectively, with moderate effects (OR>1.25). The other three regions, rs6545946 (2p15; 7.01×10^−9^), rs12677663 (8q21.11; 1.96×10^−8^) and rs11229030 (11q12.1; 8.45×10^−9^), each contained multiple candidate genes. Additionally, interrogation of publically available eQTL databases revealed that rs6545946 correlated with both *CPAMD8* and *AK3* expression [22]. Further investigation of a gap next to the 11q12.1 peak of association detected a previously reported 625 bp copy number variant (CNV) found in 1 Yoruban (YRI) HapMap sample [23], which is ∼50 kb downstream of our top SNP rs11229030. Also, in this region, 17 SNPs were filtered out due to poor imputation quality.

### Comparison of CD signals found in AJ to NJ European ancestry-derived loci

We examined LD architecture at the five novel regions in AJ and NJ CD cases from the Wellcome Trust GWAS [18] (Figure S3). We found 85 pairs of variants >150 kb apart around the top SNPs having r^2^>0.2 in AJs compared to one pair in NJs across all 5 loci. Sixty two out of the 85 linked pairs in AJs were detected at 5q21.1 versus 0 pairs in non-AJs.

To examine the established CD risks in AJ populations, we compared the signals in 71 unique susceptibility loci for CD identified in the largest meta-analysis of CD in NJ populations to date [24] to those in our sample (Table S3). We note that 57 susceptibility loci passed quality control in our analysis, of which, 31 surpassed nominal significance and 30/31 showed effects in the same direction in AJ as observed previously (p<6.98×10^−4^). We selected a subset of these 30 loci for a direct comparison of genetic variance explained at susceptibility loci in NJ and AJ. Assuming similar effect sizes in both populations, we had >80% power to detect variants conferring OR≥1.22 at the nominal significance of 0.05, assuming a minor allele frequency of >20% in healthy controls. At these thresholds, we were powered to examine signals at 12 of the known loci in the AJ sample. Of the 12 loci, 11 passed QC in our discovery panel. Greater than the nominal signal (p<0.05) was observed for 9 of the 11 loci (Table S3), which agreed with our expectation by chance (based on the power for detection, the number of signals that had been expected to attain p<0.05 is 10.15±0.86). Specifically, all 9 loci with >0.85 power to be detected were observed and altogether they explained 4.3% and 3.7% of genetic variance in AJs compared to NJs, respectively (Table S4). In all, with the three coding *NOD2* mutations, 11 confirmed SNPs (excluding the *NOD2* tagSNP rs2076756), and 5 newly-discovered variants, we can account for 11.2% of genetic contribution for CD in AJs (Table S4).

## Discussion

CD has been a forerunner for common-disease genetics, demonstrating dozens of markers associated with disease prevalence in NJ populations. Here, we report the first GWAS for CD in a sizeable increased-risk AJ population. As expected, a significant number of markers previously associated with CD in predominantly non-Jewish European cohorts were also associated with CD risk in the AJ population. That is, of the 57 loci reported in Franke et al. [24] and successfully assayed in our study, we observed nominal signal in same direction for 30 variants. Importantly, five novel loci were identified that attained genome-wide significance.

We observed genome-wide significant association with subsequent replication in a novel region on chromosome 2p15. Evidence of sizable, trans-acting eQTL effects of rs6545946 were detected, which influence *CPAMD8* (chromosome 19p13) and *AK3* (chromosome 9p24). *CPAMD8* belongs to the complement component-3/alpha-2-macroglobulin (A2M) family of proteins involved in innate immunity and damage control. Complement components recognize and eliminate pathogens by leading to direct pathogen injury or by mediating phagocytosis and intracellular killing. *CPAMD8* is expressed in a number of human tissues, including the small intestine. In response to immune stimulants, *CPAMD8* expression has been shown to be markedly up-regulated in cell culture [25]. *AK3*, or adenylate kinase, encodes a GTP:ATP phosphotransferase that is found in the mitochondrial matrix [26]. Of interest, a GWAS examining hematologic parameters identified associations to the AK3 region with platelet count and volume [27].

The GWAS and replication samples also showed combined genome-wide significant evidence for association at 8q21.11 that spans a number of genes, including *RPL7* and *KCNB2*. *RPL7*, ribosomal protein L7, has been established as an autoantigen representing a frequent target for autoantibodies from patients with systemic autoimmune diseases, such as systemic lupus erythematosus and rheumatoid arthritis [28]. The humoral autoimmune response to RPL7 apparently is driven by antigen and is T cell dependent [29]. *KCNB2* is a potassium voltage-gated channel expressed in a number of tissues, including gastrointestinal smooth muscle cells [30], [31]. Cardiac left ventricular systolic dimensions [32] and the common migraine is associated to a region that includes *KCNB2* [33].

The chromosome 11q12.1 association signal mapped to a broad region that spans multiple genes, including *SLC43A3*, *PGR2* and *PRG3*. Solute carrier family 43, member 3 (*SLC43A3*) is a putative transporter identified in a survey of microarray expression databases as having endothelial cell specific expression across multiple organs whose mRNA expression is enriched in macrophages and vascular endothelial cells [34]. Also in the region, *PGR2* and *PRG3*, proteoglycan 2 and 3, are eosinophil granule major basic proteins known as natural killer cell activators [35]. *PGR2* is believed to be involved in antiparasitic defense mechanisms as a cytotoxin and helminthotoxin, and in immune hypersensitivity reactions, including allergies and asthma [36], [37]. High levels of the proform of this protein are also present in placenta and pregnancy serum [38]. *PGR3* possesses similar cytotoxic and cytostimulatory activities to *PRG2*. In vitro, *PRG3* has been shown to stimulate superoxide production and IL8 release from neutrophils, and histamine and leukotriene C4 release from basophils [39]. Furthermore, a rare copy number variant has been reported in 1 YRI HapMap sample 34 kb downstream of the top SNP [23].

In addition, we observed genome-wide significant evidence for association on chromosome 10q26.3 that was subsequently replicated at rs10734105. This region is devoid of established coding genes and detailed functions of a single nearby gene encoding for transcription elongation regulator 1-like protein (*TCERG1L*) have not yet been reported. The most significant chromosome 5q21.1 association signal was flanked by *SLCO6A1* (solute carrier organic anion transporter family gene).

Notably, none of these novel variants have been identified by the largest CD meta-analysis of individuals of European descent [24], which was sufficiently powered to detect effect sizes reported by the present study. However, we observed substantial differences in LD architecture around the top hits across the 5 novel signals. These regions were enriched in variants >150 kb apart with moderate and high LD (r^2^>0.2) compared to individuals of European ancestry, which can, at least in part, explain the lack of signal in non-AJs. Also, existence of rare variants in these regions specific to this population cannot be ruled out.

Our data also suggest that refinement of causal alleles may increase present estimates of heritability accounted for by presently identified genetic loci. That is, the top GWAS SNP at the *NOD2* locus in AJs appears to explain 1.5% of genetic variance, whereas the three *NOD2* coding mutations themselves account for 6.1% (Table S4), which is slightly higher than in NJs (0.8% and 5%, respectively [24]). Due to the historical population bottleneck and subsequent isolation of AJs [40], it is possible that there are population-specific rare variants in the newly discovered regions contributing to CD susceptibility, reflecting allelic heterogeneity. Therefore, resequencing analysis aimed at detecting the population-specific rare variants in these regions may prove to be a more successful approach to identify functional variants associated with the disease. In all, with 19 variants, we can account for 11.2% of genetic contribution for CD in AJs.

This study brings forth some lessons from using a specific, isolated population in a large GWAS. First, as observed in other contexts, self-declared ethnicity is an imperfect indicator of genetic ancestry. Caution must be applied when considering samples purported as part of a genetically distinct population. In this study, we applied a mixed model of association, EMMAX [46], in each group separately (100%, 75%, 50% AJ, Figure 1C), thereby excluding 236 samples from analysis; of note is that among the nine previously established loci which we were powered to identify, we observed more significant evidence for association in seven of these nine loci with this grouped approach, as opposed to using a mixed model of association on the full cohort (data not shown). An additional limitation of a study in an isolated population is the availability of samples. In this case, we collected samples across multiple diseases, and rely on CD being rare enough for most of the individuals to be good controls for this disease. While the reliance on multiple cohorts from various studies exposes our study to concerns of platform-specific and center-specific artifacts, these concerns are shared by many multi-center GWAS published during the last few years. As such studies often exchanged summary statistics for meta-analysis, our study had the advantage of analyzing individual-level data at the same site and controlling their quality uniformly.

The focus on the AJ population highlights the pros and cons of conducting GWAS in a specific, isolated population versus more outbred populations. On the one hand, we observe increased detectability of some known common variants previously discovered in NJ populations in this study. That is, we observed sizable differences in the risk allele frequencies between AJ and NJ controls for some SNPs, including *IRGM* rs7714584 (16.2% vs. 8.8%) and *LRRK2* rs11564258 (5.6% versus 2.5%). While the latter can be associated with the ascertainment bias related to the inclusion of patients with Parkinson's disease as non-disease controls, the former trend was observed previously [19]. On the other, some common variants that confer CD risk in NJ populations, such as *PTPN2* and *TNFSF18*, did not replicate in the AJ panel despite sufficient power. While we assembled the largest sample of CD patients of Ashkenazi descent to date, potential explanations can include limited size, and therefore lack of power. There have been no reported sub-phenotypic differences in Crohn's disease comparing Jewish and non-Jewish cohorts. Yet, it is quite possible that different gene-environment interactions could account for the distinct genetic loci identified. In addition, our study design might have overlooked joint disease loci as many of our controls were ascertained for several complex disorders. Yet, our results follow observations in other isolated populations [41], [42] and delineate the distinct vs. shared repertoires of CD causal variants in AJs vs. NJs, in addition to population differences in patterns of LD between the causal variant and the detected marker. Resolution of the source of these differences may become available through high throughput sequencing in such samples.

Finally, looking ahead, the diversification of the population studied in SNP-based association studies is likely to become even more important with the current transition to sequencing. Population genetics theory suggests that repertoires of rare, recently-arising alleles would differ more between distinct and isolated groups. This promises increased value for isolated populations for sequencing studies that aim at dissecting the genetics of complex diseases.

## Materials and Methods

### Ethics statement

The study was approved by the Institutional Review Boards at all participating institutions, including the Mount Sinai School of Medicine, Albert Einstein College of Medicine, New York University, Hebrew University of Jerusalem, Yale University, University of Pittsburgh, Johns Hopkins University, University of Toronto, Columbia University, Tel Aviv Sourasky Medical Center, Rambam Medical Center, Cedars-Sinai Medical Center, and North Shore University Hospital-Long Island Jewish Medical Center. All patients provided written informed consent (in English or Hebrew) for the collection of samples and subsequent analysis.

### Sample collection

Participants in this study were ascertained from 11 different centers in the United States or Canada (New York, Philadelphia, Los Angeles, Pittsburgh, New Haven, Baltimore and Toronto) and Israel (Tel Aviv, Haifa and Jerusalem). In total, 6,370 individuals who self-identified as AJ participated in the study. Blood samples were taken with informed consent for DNA extraction. Standard criteria that were used for the diagnosis of Crohn's disease (CD) at each center included the characteristic symptoms of chronic duration and objective validation, including endoscopic, radiologic and/or pathologic confirmation [43].

The initial discovery GWAS analysis combined raw genotype data obtained from genome-wide screening arrays across five studies. The combined discovery AJ GWAS sample consisted of 907 CD cases and 2,345 “controls”, where the control population was made up of individuals ascertained as non-Crohn's disease (non-CD) cases (AJ individuals with Parkinson's disease, Schizophrenia, Type-2 Diabetes and Dystonia) or AJ individuals ascertained as non-diseased controls (1,006 and 1,339, respectively) (Table 1).

An independent AJ replication sample was used to validate findings from the discovery GWAS. These included samples that had been genotyped both on large-scale platforms and on custom arrays. The final replication cohort consisted of 623 CD cases and 2,124 controls of AJ descent (565 and 1,559 non-CD cases and non-disease controls, respectively). For a subset of 31 replication markers, we included an extra 348 AJ cases genotyped using the Illumina 610 k array. Details of all cases and controls genotyped and the genotyping platforms used are given in Table 1.

### Quality control (QC) measures for combining multiple genome-scale datasets

We devised a strategy to combine the raw genotypes from nine separate genome-scale datasets of variable size (59–1,067 individuals) and case:control composition, that were genotyped across several different platforms (Illumina 300 k, 500 k, 660 k and 1 M and Affymetrix 500 k and 6.0) (see Text S1 for details). All of the analyses were performed in PLINK [44]. The combined analysis QC pipeline is shown in Figure S1.

### AJ ancestry verification

PCA was conducted with smartpca software [45] using the intersection of markers typed on all Illumina and Affymetrix platforms in the combined dataset. We trained a coordinate system across the ∼22 K unlinked SNPs in the sample, including the three continental Hapmap populations (Yoruban (YRI, n = 167), combined Han Chinese and Japanese (CHB, n = 84 and JPT, n = 86) and European (CEU, n = 164)) and populations from the Jewish Hapmap [2] of Middle Eastern Jews (Iraqi (n = 40), Iranian (n = 32) and Syrian Jews (n = 25)), and European origin Jews (Italian (n = 39), Ashkenazi (n = 35) and Sephardic Jews from Greece (n = 44) and Turkey (n = 34)) (Figure 1A). The analysis was repeated excluding the YRI, CHB and JPT samples. Ancestry for all participants in the study was assessed by PCA projection of their genotypes onto coordinates derived from training on the reference panels. Individuals that clustered distinctly with the Ashkenazi reference panel were deemed to have 100% AJ ancestry (group 1) (Figure 1B). In addition, two other groups of individuals that were intermediate between the Ashkenazi and CEU reference panel clusters were included in the subsequent analysis; individuals with 75% AJ:25% European ancestry and 50% AJ:50% NJ, groups 2 and 3, respectively (Figure 1C). Samples that fell outside group 1–3 modes as determined by PCA analysis, were excluded from the study (n = 236) (Table S1 and Text S1).

### Constructing an AJ reference panel

Due to concerns over poor quality for imputed genotypes in AJ samples using any of the standard HapMap reference panels, we constructed a population-specific AJ reference panel comprised of 100 AJ individuals who had been typed on both the Affymetrix 6.0 and Illumina Omni1 platforms (see Figure S4 and Text S1).

### Discovery GWAS

After cleaning and pruning for ancestry, the discovery GWAS comprised a total of 2,994 participants, 737 CD cases and 2,257 controls. The discovery GWAS population was divided according to AJ ancestry groups (Figure 1C). The final counts of CD cases/controls in each group were: group 1 (100% AJ) 632/2,107, group 2 (75% AJ) 36/38 and group 3 (50% AJ) 69/212.

AJ populations are known to exhibit a high degree of cryptic relatedness relative to outbred populations [5], therefore we selected a mixed-model method for association, EMMAX, that could account for any residual substructure of the AJ population [46]. We tested for association to CD in each group separately. To test for over-dispersion in the presence of strong effects, we repeated the analysis excluding the top 7 *NOD2* SNPs. Any over-inflation of the p-value distributions was adjusted by genomic control to approximate normality uniform p-value distribution [20]. P-values were combined across the three groups using METAL [47] (Text S1).

### Replication

A total of 175 markers were selected for replication (Table S2). The replication dataset consisted of participants with (a) confirmed AJ ancestry genotyped on genome-scale Affymetrix and Illumina platforms from QC-filtered cohorts which had not been included in the discovery GWAS (n = 929) and (b) self-reported AJ ancestry genotyped on custom Sequenom iPlex arrays (n = 1,841) (Table 1). For a subset of replication markers (n = 31), we included additional set (c) of CD cases with AJ ancestry identified by PCA and genotyped on the Illumina 610 k platform (n = 348) (Table 1).

The direction of effect of markers surpassing nominal significance in the replication dataset was compared between both the discovery and replication datasets and markers that had opposite effects were excluded. The one-tailed p-value of replicating markers was then combined with the discovery p-value using Fisher's combined p-value method to produce the per-SNP combined score.

### Comparison to known European ancestry hits

Risk alleles and direction of effect were compared in both NJ and AJ samples for concordance. Power calculations were performed using the Genetic Power Calculator [48]. We also compared LD architecture 250 kb upstream and downstream of the novel hits between AJs and NJs using 1,748 CD cases of European ancestry from the Wellcome Trust GWAS [18] by assessing the number of SNP pairs located far apart with various levels of linkage disequilibrium. Fraction of genetic variance explained by the top risk alleles was assessed using the liability threshold model of Risch [49] considering contributions to be additive. The calculations were based on a prevalence of Crohn's disease in AJs of 1 per 100. For the coding *NOD2* variants, we used previously reported frequencies and effect sizes [19].

## Supporting Information

Figure S1 Schema of combined analysis of discovery GWAS dataset. Illumina (n = 3) and Affymetrix (n = 3) raw genotypes from the different self-reported Ashkenazi (AJ) cohorts were quality control filtered before being combined in three groups; Group 1 = 100% AJ, Group 2 = 75% AJ: 25% Non-Jewish European (NJ) and Group 3 = 50% AJ:50%NJ. Missing variants within each group were imputed from a specially constructed AJ reference panel comprising 98 individuals sequenced on both Affymetrix and Illumina platforms. Each group was then filtered for low imputation score and minor allele frequency, and for batch effects between platforms and cohorts, to yield the final GWAS discovery datasets.
(DOC)

Figure S2 Regional plots of known Crohn's disease loci in Ashkenazi Jews. Regional plots of the SNP p-values obtained in the discovery GWAS for a ±250 kb window around each of the 5 novel SNPs. The X-axis shows the chromosome and physical distance (kb), the left Y-axis shows the negative base ten logarithm of the p-value and the right y-axis shows recombination activity (cM/Mb) as a blue line. The chromosomal band is given above each plot. The replication SNP is indicated as a large red diamond, and linkage disequilibrium of surrounding SNPs with the replication SNP is indicated by a scale of intensity of red color filling as shown in the legend at the upper right hand corner of each plot. The combined discovery and replication p-value for the replication SNP is shown in blue, and is annotated with the SNP identifier and combined p-values. Positions, recombination rates and gene annotations are according the NCBI's build 36 (hg 18).
(DOC)

Figure S3 Comparison of LD architecture between 100% AJ CD cases and NJ CD cases (from WTCCC [18]) at 5 novel regions of association from this study. Plots of linkage disequilibrium of in a ±250 kb window around each of the 5 novel SNPs in 100% AJ CD cases (n = 638) and European ancestry NJ cases from the WTCCC [18] (n = 1,748).
(DOC)

Figure S4 Concordance between Illumina and Affymetrix platforms Concordance was determined between individuals (n = 100) and SNPs (n∼195 K) that were genotyped on both the Affymetrix 6.0 and Illumina 1 M platforms in the reference panel (A) Shows the cumulative concordance between SNPs, where the grey bar shows the cut off for inclusion in the reference panel and (B) Shows the concordance per individual, where two individuals with <99.7% concordance were excluded.
(DOC)

Table S1 Ashkenazi Jewish ethnicity of study participants in discovery GWAS cohorts. For each cohort (Data Source) in the discovery GWAS, the total number of participants is shown. Individuals with 100% Ashkenazi ancestry or either 75%∶25% or 50%∶50% Ashkenazi∶European ancestry are shown (A J_100_, AJ_75_ and AJ_50_) as revealed by PCA analysis comparing these samples to representative groups of European Ancestry (HapMap CEU) and non-Ashkenazi Jewish Ancestry individuals (JHapMap [2]). Individuals with <50% Ashkenazi Jewish ancestry and/or non-Ashkenazi Jewish ancestry are shown (Others) and were excluded from the subsequent analysis.
(DOC)

Table S2 175 SNPs selected for replication. Replication Region #: a region for replication containing ≥1 tag SNP (each region is also banded alternatively with white or blue color fill); dbSNP identifier: the unique rs identifier for each SNP (dbSNP 130/hg18); Chromosome and Physical position: the chromosomal and physical position of each SNP (hg18); Novel or Known region?; if variants the region has been previously associated with risk for CD in Franke *et. al* 2010 [24], noteworthy gene(s) in the region are listed, otherwise the region is indicated as “Novel”; Discovery GWAS p-value; reported p-value in the AJ panel for the discovery phase of this study; Minor allele; minor allele in the AJ panel; Frequency of the minor allele; frequency of the minor allele in all CD cases and controls; frequency is also given for CD cases, non-CD disease controls and controls stratified by cohort; Passed replication assay?; whether the SNPs was successfully genotyped on the Sequenom platform; Replication p-value; the p-value for association to CD in the replication panel; Replicated p<0.05; yes if the replication p-value<0.05; Combined p-value; the combined p-value for association for SNPs that passed replication via Fishers combined probability test of the discovery GWAS and replication p-values; SCAN-P; p-value for the same SNP in the discovery meta-analysis from the Franke *et. al.* study [24].
(XLS)

Table S3 Comparison of frequency, odds ratio and p-value in the AJ panel for 71 SNPs associated to CD in mainly European ancestry individuals in Franke *et. al.* 2010 [24]. SNP: the unique rs identifier for each SNP (dbSNP 130/hg18); Chromosome and Physical position: the chromosomal and physical position of each SNP (hg18); Risk allele; the reported risk allele from the Franke *et. al.* study in both populations; Freq, OR, P-value; comparing the Non-Jewish individuals from Franke *et al.* [24] (NJ) to the Ashkenazi panel reported here (AJ) given the frequency, odds ratio and p-values for each of the 71 SNPs from the meta-analysis; effect in same direction?; if the odds ratio for the reported risk allele is in the same direction the “yes”, otherwise, “no”; AJ nominal (p<0.05)?; if the discovery GWAS p-value (or replication p-value where the associated SNP was the same in both studies) is less than p<0.05 then “yes”, otherwise, “no”; Noteworthy genes; interesting genes in the mapping interval for association. Assuming similar effect sizes, we had >80% power to detect variants conferring OR≥1.22 at the nominal significance of 0.05, assuming a minor allele frequency of >20% in healthy controls. At these thresholds, we were powered to examine signals at 12 of the known loci in the AJ sample (indicated as bolded rows in the spreadsheet). Of the 12 loci, 11 were assayed in our discovery panel. Greater than the nominal signal (p<0.05) was observed for 9 of the 11 loci which were then used for direct comparison of signal for association to CD between NJ and AJ panels.
(XLS)

Table S4 Calculation of variance explained for 17 replicated and associated SNPs from this study and 9 SNPs used for comparison of NJ versus AJ signal at known associated loci. The fraction of genetic variance explained was calculated for 17 replicated and associated SNPs from this study and 9 SNPs used for comparison of NJ vs AJ, using the liability threshold model given in Risch *et al.* [49], assuming an additive effect. We also assumed a prevalence of CD in NJs to be 0.4% and 1% in AJs. The calculation is coded as a macro in the excel spreadsheet (columns B through AC).
(XLS)

Text S1 Supplemental material.
(DOC)
