# Supplementary figures and images for: A Genome-Wide Scan of Ashkenazi Jewish Crohn's Disease Suggests Novel Susceptibility Loci
Source: PLoS Genet. 2012 Mar 8;8(3):e1002559. doi: 10.1371/journal.pgen.1002559 (PMC3297573; doi:10.1371/journal.pgen.1002559)

Figure S1


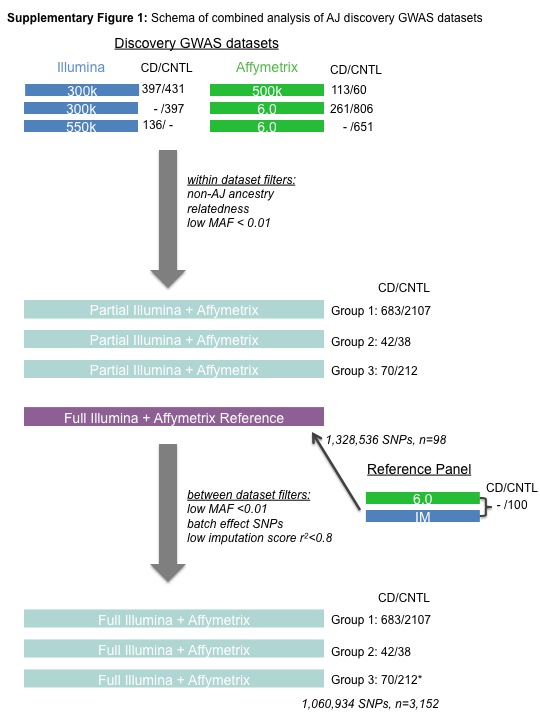

Supplement: Figure S1 — Schema of combined analysis of discovery GWAS dataset. Illumina (n = 3) and Affymetrix (n = 3) raw genotypes from the different self-reported Ashkenazi (AJ) cohorts were quality control filtered before being combined in three groups; Group 1 = 100% AJ, Group 2 = 75% AJ: 25% Non-Jewish European (NJ) and Group 3 = 50% AJ:50%NJ. Missing variants within each group were imputed from a specially constructed AJ reference panel comprising 98 individuals sequenced on both Affymetrix and Illumina platforms. Each group was then filtered for low imputation score and minor allele frequency, and for batch effects between platforms and cohorts, to yield the final GWAS discovery datasets. (DOC) [file pgen.1002559.s001.doc]

**Figure S2. Regional plots of known Crohn’s disease loci in Ashkenazi Jews.**
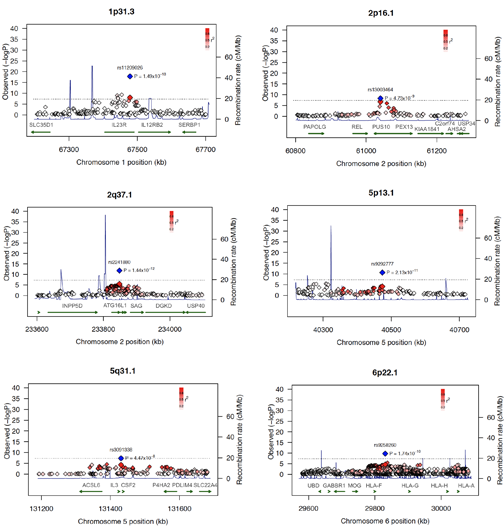


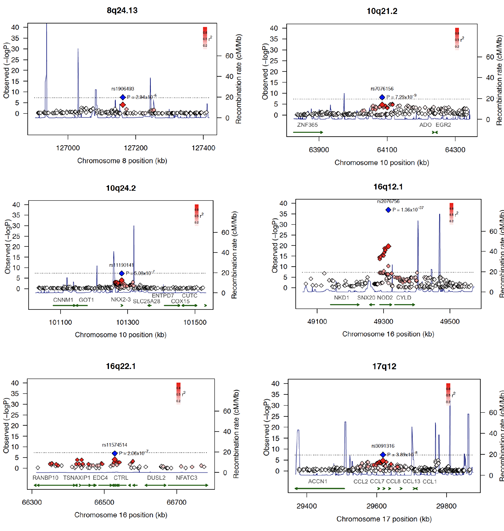

Supplement: Figure S2 — Regional plots of known Crohn's disease loci in Ashkenazi Jews. Regional plots of the SNP p-values obtained in the discovery GWAS for a ±250 kb window around each of the 5 novel SNPs. The X-axis shows the chromosome and physical distance (kb), the left Y-axis shows the negative base ten logarithm of the p-value and the right y-axis shows recombination activity (cM/Mb) as a blue line. The chromosomal band is given above each plot. The replication SNP is indicated as a large red diamond, and linkage disequilibrium of surrounding SNPs with the replication SNP is indicated by a scale of intensity of red color filling as shown in the legend at the upper right hand corner of each plot. The combined discovery and replication p-value for the replication SNP is shown in blue, and is annotated with the SNP identifier and combined p-values. Positions, recombination rates and gene annotations are according the NCBI's build 36 (hg 18). (DOC) [file pgen.1002559.s002.doc]

**Figure S4.** **Concordance between Illumina and Affymetrix platforms**


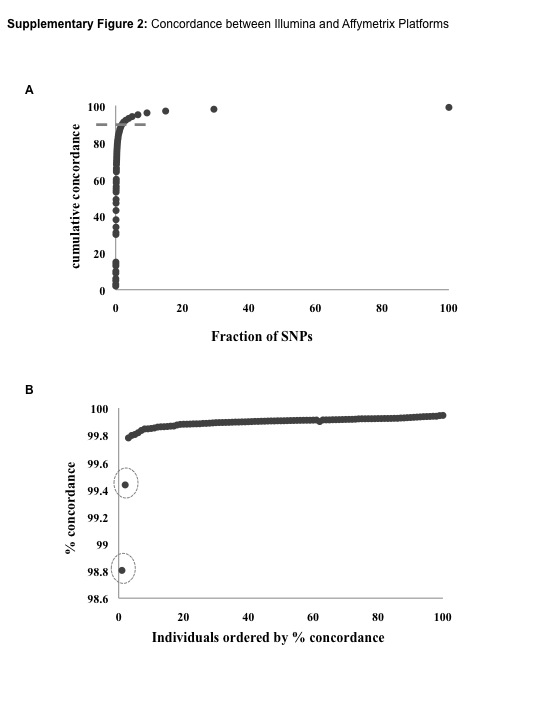

Supplement: Figure S4 — Concordance between Illumina and Affymetrix platforms Concordance was determined between individuals (n = 100) and SNPs (n∼195 K) that were genotyped on both the Affymetrix 6.0 and Illumina 1 M platforms in the reference panel (A) Shows the cumulative concordance between SNPs, where the grey bar shows the cut off for inclusion in the reference panel and (B) Shows the concordance per individual, where two individuals with <99.7% concordance were excluded. (DOC) [file pgen.1002559.s004.doc]
