# Supplementary material for: A Genome-Wide Scan of Ashkenazi Jewish Crohn's Disease Suggests Novel Susceptibility Loci
Source: PLoS Genet. 2012 Mar 8;8(3):e1002559. doi: 10.1371/journal.pgen.1002559 (PMC3297573; doi:10.1371/journal.pgen.1002559)
Supplement: Figure S3 — Comparison of LD architecture between 100% AJ CD cases and NJ CD cases (from WTCCC [18]) at 5 novel regions of association from this study. Plots of linkage disequilibrium of in a ±250 kb window around each of the 5 novel SNPs in 100% AJ CD cases (n = 638) and European ancestry NJ cases from the WTCCC [18] (n = 1,748). (DOC) [file pgen.1002559.s003.doc]

**Figure S3. Comparison of LD architecture between 100% AJ CD cases and NJ CD cases (from WTCCC^8^) at 5 novel regions of association from this study.**

| **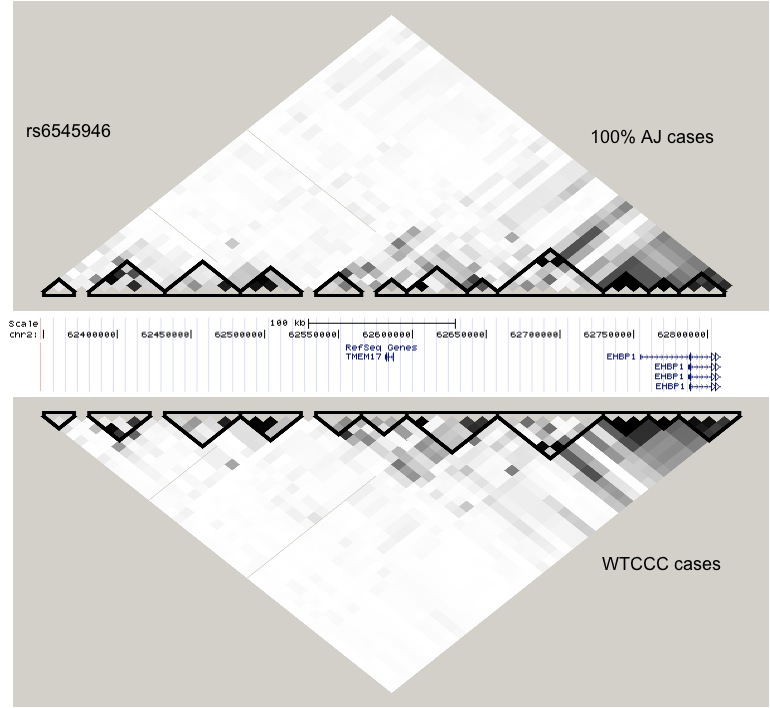** |
| --- |

| **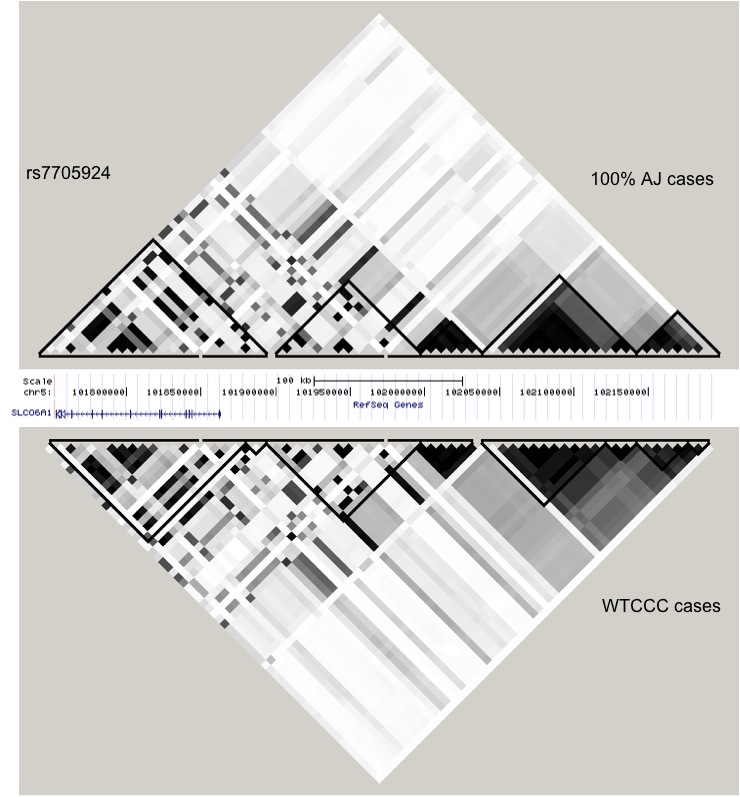** |
| --- |

| **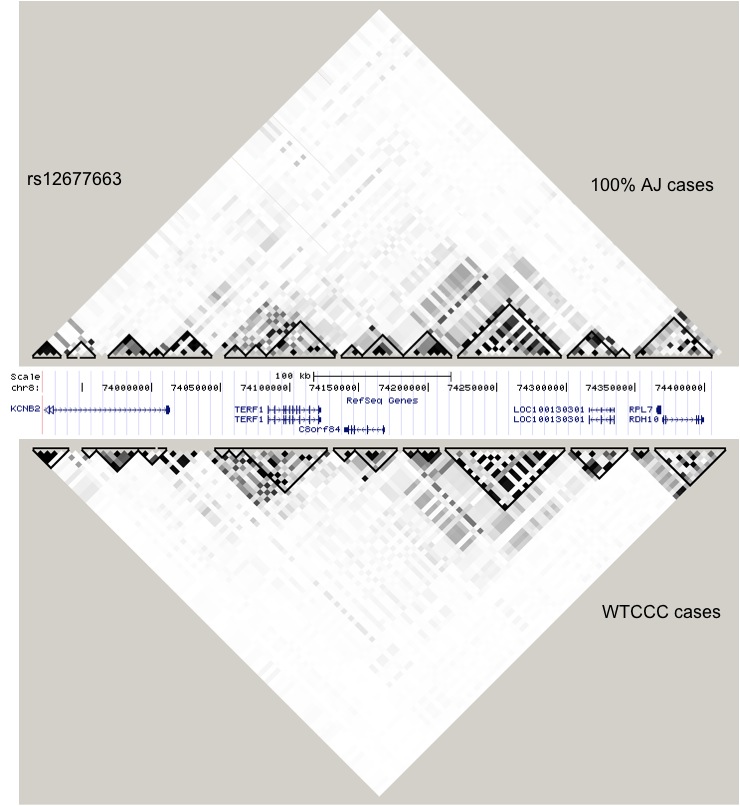** |
| --- |

| **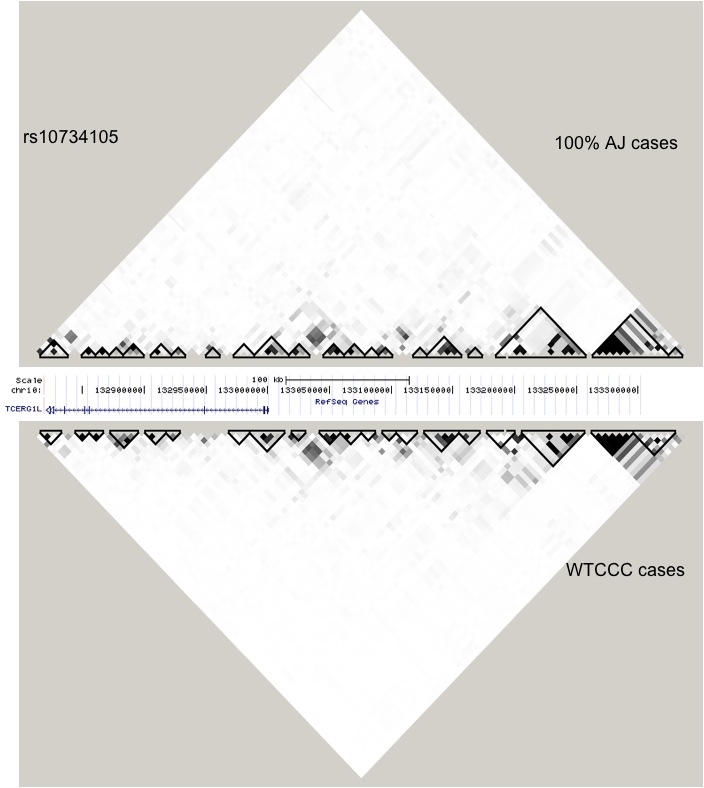** |
| --- |

| **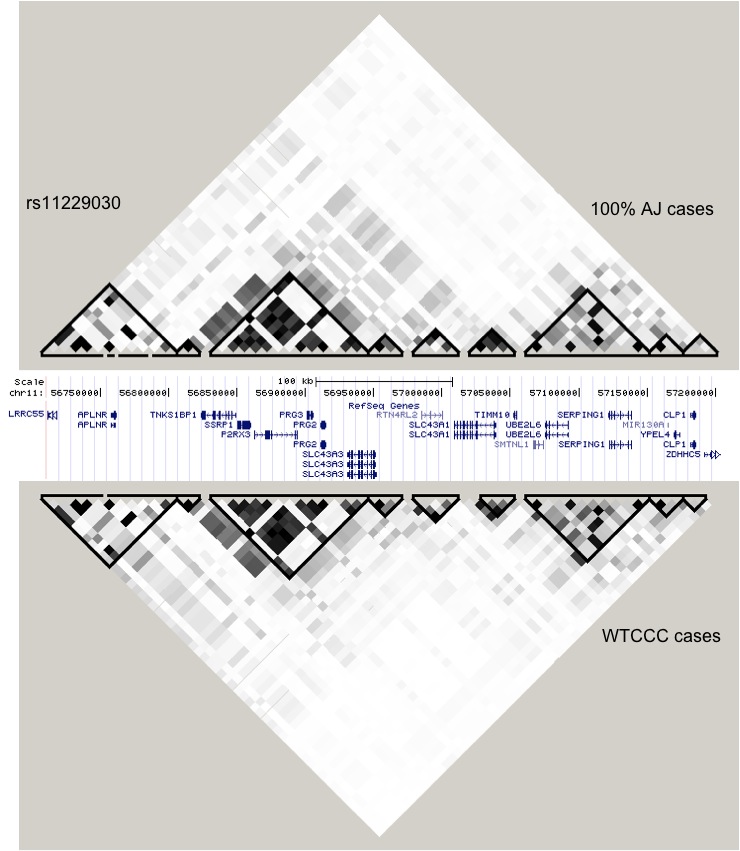** |
| --- |
