# Supplementary material for: A Genome-Wide Scan of Ashkenazi Jewish Crohn's Disease Suggests Novel Susceptibility Loci
Source: PLoS Genet. 2012 Mar 8;8(3):e1002559. doi: 10.1371/journal.pgen.1002559 (PMC3297573; doi:10.1371/journal.pgen.1002559)
Supplement: Table S1 — Ashkenazi Jewish ethnicity of study participants in discovery GWAS cohorts. For each cohort (Data Source) in the discovery GWAS, the total number of participants is shown. Individuals with 100% Ashkenazi ancestry or either 75%∶25% or 50%∶50% Ashkenazi∶European ancestry are shown (A J100, AJ75 and AJ50) as revealed by PCA analysis comparing these samples to representative groups of European Ancestry (HapMap CEU) and non-Ashkenazi Jewish Ancestry individuals (JHapMap [2]). Individuals with <50% Ashkenazi Jewish ancestry and/or non-Ashkenazi Jewish ancestry are shown (Others) and were excluded from the subsequent analysis. (DOC) [file pgen.1002559.s005.doc]

**Table S1:** Ashkenazi Jewish ethnicity of study participants in discovery GWAS cohorts

| **Data Source** | **Samples** | **AJ_100_** | **AJ_75_** | **AJ_50_** | **Other** | **AJ_total_** |
| --- | --- | --- | --- | --- | --- | --- |
| NIDDK IBD genetics consortium | 828 | 702 | 30 | 37 | 59 | **769** |
| Pediatrics IBD Consortium | 136 | 66 | 12 | 31 | 27 | **109** |
| Mount Sinai School of Medicine I | 173 | 156 | 9 | 4 | 4 | **169** |
| Mount Sinai School of Medicine II | 532 | 412 | 9 | - | 111 | **421** |
| John Hopkins University | 535 | 529 | 5 | - | 1 | **534** |
| Albert Einstein College of Medicine | 651 | 629 | 7 | - | 15 | **636** |
| Hebrew University of Jerusalem | 397 | 367 | 2 | 9 | 19 | **378** |
| **Total** | **3,252** | **2,861** | **74** | **81** | **236** | **3,016** |
