# Supplementary material for: A Genome-Wide Scan of Ashkenazi Jewish Crohn's Disease Suggests Novel Susceptibility Loci
Source: PLoS Genet. 2012 Mar 8;8(3):e1002559. doi: 10.1371/journal.pgen.1002559 (PMC3297573; doi:10.1371/journal.pgen.1002559)
Supplement: Text S1 — Supplemental material. (DOC) [file pgen.1002559.s009.doc]

**Supplemental Material**

*Quality control measures for combining multiple genome-scale datasets*

We devised a careful strategy to combine the raw genotypes from nine separate genome-scale cohorts of variable size (59-1,067 individuals) and case:control composition, that were genotyped across several different platforms (Illumina 300k, 500k, 610k, 660k, and 1M and Affymetrix 500k and 6.0). All following analyses were performed in PLINK. First we applied stringent quality control measures to each of the datasets independently. Homozygous heterozygote SNPs were set to missing (--set-hh-missing), genotypes out of Hardy-Weinberg equilibrium were excluded (--hwe 0.001) and each dataset was filtered for genotypes with >0.01 missingness (--geno 0.01) or samples with >0.01 missing genotypes (--mind 0.01). Where duplicates samples within and between datasets were detected (n=9), using PLINK to detect excess allele sharing (--genome, >0.9), one of the pairs was removed. We also excluded genotypes with minor allele frequencies < 0.01 (--maf 0.01). Before merging the raw genotypes, we detected and resolved any conflicting genotype annotation or individual identifiers between datasets. We also excluded a subset of “ambiguous” genotypes from the Affymetrix platforms – genotypes that are A/T or C/G, since, when merging, it is difficult to determine if these genotypes are “flipped” (the major allele in one dataset is the minor allele in another) or annotated on opposite strands between datasets. Merging (--merge) yielded a combined dataset of partial genotyping across markers from both Affymetrix and Illumina platforms.

To detect spurious associations due to batch-, cohort- and/or non-CD disease-specific effects, the merged dataset was stratified by case or control status and by Illumina or Affymetrix platform. Under a null hypothesis of no batch effects, the p-values of a case vs. case or control vs. control association should be uniformly distributed. Therefore, we tested for batch effects by analyzing association between sub-categories of control vs. controls: (a) platform specific effects with controls on Illumina vs. controls on Affymetrix platforms, (b) cohort specific effects with controls from one cohort vs. controls from another cohort genotyped on the same platform (c) non-CD disease effects by controls vs. non-CD disease by cases from the same cohort. We repeated the same analysis using cases vs. cases. We selected for non-uniformly distributed p-values using a conservative threshold of 1x10^-4^ as some of the sub-categories we tested were low powered due to small numbers. A total of 5,935 markers were excluded. Finally, it has been previously shown that markers at the *LRRK2* and *GBA* loci show strong association to Parkinson’s disease in AJ, we excluded all markers at those loci (n= 395).^1^ Combined analysis quality control pipeline is shown in **Supplemental Figure 1.**

*AJ ancestry verification*

PCA was conducted with smartpca software ^2^ and default settings with no outliers removed. We first identified the intersection of markers typed on all Illumina and Affymetrix platforms in the combined dataset of genotyped markers (n=~27K). We then used PLINK to reduce linkage disequilibrium between markers (--indep-pairwise 50 5 0.3) by removing all markers with r^2^>0.3 (n=~4K). Since extended linkage disequilibrium is known to confound PCA ^3^, we also excluded markers in regions of known strong association to CD and Parkinson’s disease in AJ populations (*LRRK2*, *GBA*, *NOD2* and *IL23R* gene region and the HLA region) and the markers flanking centromeres (n=~1k). NJ or known AJ ancestry samples that fell outside group 1-3 modes as determined by PCA analysis, were excluded from the study (n=236).

### Constructing an AJ reference panel

Due to concerns over poor imputation quality for imputed genotypes in AJ samples using any of the standard HapMap reference panels, we constructed an AJ reference panel comprised of 100 AJ individuals who had been typed on both the Affymetrix 6.0 and Illumina 1M platforms, resulting in a combined 1,778,360 raw genotypes. Genotypes with >0.02 missingness, only one allele called or low frequency (MAF <0.02) were excluded from the reference panel. Ambiguous SNPs (A/T or C/G) on the Affymetrix platform were also excluded before merging (see previous section). Finally, we examined genotype concordance in the subset of markers called by both the Affymetrix and Illuminia platforms in the reference panel individuals. A total of 319 markers were ≥ 0.1 discordant between platforms, which represent a small fraction (0.0164) of overlapping markers in the subset, a projected <0.001 discordance between all markers from both platforms in the reference panel (**see Supplemental Figure 4A**). Two individuals who were outliers for discordance (>0.008) were removed from the reference panel and the average “between platform” concordance was >0.997 in the remaining individuals (**see Supplemental Figure 4B**). Missing markers within the reference panel were imputed using BEAGLE.^4^ BEAGLE’s output includes a posterior probability score (0-1) for any inferred allele. Imputed markers with scores < 0.9 were excluded from the reference panel. The final panel consisted of 1,328,536 genotypes in 98 individuals (**see Supplemental Figure 1)**.

### Imputing missing alleles in the combined dataset

Missing genotypes in the combined AJ dataset were imputed by training on the AJ reference panel using BEAGLE ^4^ using a posterior probability score (0-1) for any inferred allele. Since the EMMAX method is limited to exact allele calls, we took a conservative approach to threshold for high quality imputed genotype calls based on r^2^>0.8 in order to minimize false positives. Inferred genotypes with low frequency (MAF <0.01) were excluded from the subsequent analysis. The final combined reference dataset contained 1,060,934 SNPs.

## *Discovery GWAS*

After cleaning and pruning for ancestry, the discovery GWA comprised a total of 2,994 participants, 737 CD cases and 2,257 controls. The discovery GWAS population was divided according to AJ ancestry groups (**Figure 1C**). We noted that group 3 (50% AJ ancestry) contained ~3-fold more CD cases than controls and thus would be underpowered for detecting a CD-driven signal. To optimize the power in group 3, and under the assumption that group 3 comprised individuals that were 50% AJ/50% NJ, we added 100 randomly drawn controls removed from group 1 and 100 randomly select NJ controls from the HapMap CEU population to group 3. The final counts of CD cases/controls in each group were: group 1 (100% AJ) 632/2,107, group 2 (75% AJ) 36/38 and group 3 (50% AJ) 69/212.

We tested for association to CD in each group separately using EMMAX ^5^. We first built a kinship matrix of pair-wise identity-by-state metrics based on the high-density markers for each group using EMMAX default settings (-v –h –s –d 10). Each kinship matrix was then added to the mixed-model for association mapping of CD cases vs. controls for the respective groups (-v -d 10 -k [kinship file]), which resulted in a p-value of association for each marker per group. To test for over-dispersion in the presence of strong effects, we repeated the analysis excluding the top 7 *NOD2* SNPs. Any over-inflation of the p-value distributions was adjusted by genomic control to approximate normality uniform p-value distribution ^6^. P-values were then combined across the three groups using METAL ^7^, which takes direction of effect into account and weights the groups by sample size, to produce the final mapping p-values for the discovery GWAS.

*Replication*

A total of 188 markers were selected for replication (see results for selection criteria and **Supplemental Table 2**). PCR primers and extension primers for each SNP were designed by the software MassARRAY Assay Design (Sequenom, Inc). Two SNPs failed the assay design and 186 SNPs were plexed into five custom Sequenom iPlex assays. In all, 147 SNPs were successfully genotyped. Eight SNPs twice failed plex attempts and eight others had < 90 % genotyping call rates by the Sequenom iPlex. Two major histocompatibility complex (MHC) SNPs, rs404860 and rs4713630, that failed the Sequenom assays were genotyped using validated TaqMan® SNP genotyping assays according to manufacturer’s protocol (Applied Biosystems).

We screened for duplicate participants within and between the replication datasets. Where duplicates were detected (as having a
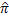
> 0.9, n=37), one sample was removed from each duplicate pair. Duplicates between the replication dataset and the discovery dataset (n=15) were also removed from the replication dataset. First and second degree relatedness was detected in the genome-scale QC-filtered dataset and related individuals were removed, preferentially keeping CD cases (n=5). However, it should be noted that familial relationships could not be confidently estimated at the resolution of 147 and 31 markers in the iPlex and Illumina 610k datasets, respectively. The final combined replication panel comprised 2,713 individuals (643 CD cases and 2,070 controls) across 147 markers, with additional 348 CD cases for a subset of 31 markers. Association mapping to CD in the replication panel was performed by the χ^2^ test in PLINK (--assoc). Direction of effect of markers surpassing nominal significance in the replication dataset was compared between both the discovery and replication datasets and markers that had opposite effects were excluded. The one-tailed p-value of replicating markers was then combined with the discovery p-value using Fisher’s combined p-value method to produce the per-SNP combined score.

**References:**

1. Thaler, A., Ash, E., Gan-Or, Z., Orr-Urtreger, A., and Giladi, N. (2009). The LRRK2 G2019S mutation as the cause of Parkinson's disease in Ashkenazi Jews. J Neural Transm 116, 1473-1482.

2. Price, A.L., Patterson, N.J., Plenge, R.M., Weinblatt, M.E., Shadick, N.A., and Reich, D. (2006). Principal components analysis corrects for stratification in genome-wide association studies. Nat Genet 38, 904-909.

3. Auton, A., Bryc, K., Boyko, A.R., Lohmueller, K.E., Novembre, J., Reynolds, A., Indap, A., Wright, M.H., Degenhardt, J.D., Gutenkunst, R.N., et al. (2009). Global distribution of genomic diversity underscores rich complex history of continental human populations. Genome Res 19, 795-803.

4. Browning, S.R., and Browning, B.L. (2007). Rapid and accurate haplotype phasing and missing-data inference for whole-genome association studies by use of localized haplotype clustering. Am J Hum Genet 81, 1084-1097.

5. Kang, H.M., Sul, J.H., Service, S.K., Zaitlen, N.A., Kong, S.Y., Freimer, N.B., Sabatti, C., and Eskin, E. Variance component model to account for sample structure in genome-wide association studies. Nat Genet 42, 348-354.

6. Devlin, B., and Roeder, K. (1999). Genomic control for association studies. Biometrics 55, 997-1004.

7. Willer, C.J., Li, Y., and Abecasis, G.R. (2010). METAL: fast and efficient meta-analysis of genomewide association scans. Bioinformatics 26, 2190-2191.

8. Consortium, W.T.C.C. (2007). Genome-wide association study of 14,000 cases of seven common diseases and 3,000 shared controls. Nature 447, 661-678.

9. Atzmon, G., Hao, L., Pe'er, I., Velez, C., Pearlman, A., Palamara, P.F., Morrow, B., Friedman, E., Oddoux, C., Burns, E., et al. (2010). Abraham's Children in the Genome Era: Major Jewish Diaspora Populations Comprise Distinct Genetic Clusters with Shared Middle Eastern Ancestry. Am J Hum Genet.

10. Franke, A., McGovern, D.P., Barrett, J.C., Wang, K., Radford-Smith, G.L., Ahmad, T., Lees, C.W., Balschun, T., Lee, J., Roberts, R., et al. (2010). Genome-wide meta-analysis increases to 71 the number of confirmed Crohn's disease susceptibility loci. Nat Genet 42, 1118-1125.

11. Risch, N.J. (2000). Searching for genetic determinants in the new millennium. Nature 405, 847-856.
